# 2126. Safety, Tolerability, and Pharmacokinetics (PK) in Healthy Participants Following Single Dose Administration of Zosurabalpin, a Novel Pathogen-Specific Antibiotic for the Treatment of Serious *Acinetobacter* Infections

**DOI:** 10.1093/ofid/ofad500.1749

**Published:** 2023-11-27

**Authors:** Andreas Guenther, Laurie Millar, Amanda Messer, Mylène Giraudon, Katie Patel, Erik J Deurloo, Michael Lobritz, Andreas Gloge

**Affiliations:** ​F. Hoffmann-La Roche Ltd, Basel, Basel-Stadt, Switzerland; Roche Ltd, Welwyn Garden City, England, United Kingdom; Roche Products Limited, Welwyn Garden City, England, United Kingdom; F. Hoffmann-La Roche Ltd, Basel, Basel-Stadt, Switzerland; Roche Products Limited, Welwyn Garden City, England, United Kingdom; F. Hoffmann-La Roche Ltd, Basel, Basel-Stadt, Switzerland; Roche, Basel, Basel-Stadt, Switzerland; F. Hoffmann-La Roche, Basel, Basel-Stadt, Switzerland

## Abstract

**Background:**

Zosurabalpin is the first representative of a novel class of tethered macrocyclic peptide antibiotics active against *Acinetobacter* spp., including carbapenem-resistant *acinetobacter baumannii-calcoaceticus* (ABC) complex organisms. This presentation summarizes and discusses unpublished preliminary safety and PK data of zosurabalpin from two completed Phase 1 clinical trials.

**Methods:**

The entry-into-human study (BP41732) was a double-blind, placebo-controlled, randomized, single-and multiple ascending dose study in healthy participants. In Part 1 (SAD) of Study BP41732, participants were randomized 3:1 to receive IV zosurabalpin or placebo in sequential single ascending dose cohorts. The mass balance study (BP43532) was a non-randomized, non-controlled, open-label, single IV dose study in healthy male participants.

**Results:**

Across these studies, 64 healthy male and female participants received a single IV dose of zosurabalpin ranging from 10 mg to 2000 mg. Participants receiving zosurabalpin, did not show any clinically significant changes compared to placebo in laboratory values, ECGs or vital signs compared to placebo.

The most common treatment-related adverse events were infusion-related reactions (IRRs), (9/64). IRR occurrence appeared to be dose-dependent, and all IRRs were fully reversible and mostly of mild intensity (7 mild/ 2 moderate).

The observed plasma exposures (C_max_ and AUC_inf_) of zosurabalpin increased approximately dose-proportionally up to 1000 mg, and more than dose-proportionally thereafter. Total [^14^C]-radioactivity was approximately equally excreted in urine and feces, with on average 52.6% and 48.0% of the administered dose recovered, respectively.

**Conclusion:**

Single IV doses of 10 mg to 2000 mg zosurabalpin were safe and overall well tolerated, and displayed a well-behaved PK profile in healthy participants. These data support evaluation of repeat dose administration in healthy participants.

**Disclosures:**

**Andreas Guenther, PhD**, ​F. Hoffmann-La Roche Ltd: Employee **Amanda Messer, BPharm**, Roche Products Ltd: Employee **Mylène Giraudon, PharmD**, F. Hoffmann-La Roche Ltd: Employee **Katie Patel, MSc**, Roche Products Limited: Employer|Roche Products Limited: Stocks/Bonds **Erik J. Deurloo, MSc**, F. Hoffmann-La Roche Ltd: Salary|F. Hoffmann-La Roche Ltd: Stocks/Bonds **Michael Lobritz, MD, PhD**, Roche: full time employee|Roche: full time employee

